# The molecular evolution of *PL10 *homologs

**DOI:** 10.1186/1471-2148-10-127

**Published:** 2010-05-03

**Authors:** Ti-Cheng Chang, Wan-Sheng Liu

**Affiliations:** 1 Department of Dairy and Animal Science, The Center for Reproductive Biology and Health (CRBH), College of Agricultural Sciences, The Pennsylvania State University, University Park, PA, 16802, USA; 2 The Integrative Biosciences Program, Bioinformatics and Genomics Option, The Huck Institute of Life Sciences, The Pennsylvania State University, University Park, PA, 16802, USA

## Abstract

**Background:**

*PL10 *homologs exist in a wide range of eukaryotes from yeast, plants to animals. They share a DEAD motif and belong to the DEAD-box polypeptide 3 (*DDX3*) subfamily with a major role in RNA metabolism. The lineage-specific expression patterns and various genomic structures and locations of *PL10 *homologs indicate these homologs have an interesting evolutionary history.

**Results:**

Phylogenetic analyses revealed that, in addition to the sex chromosome-linked *PL10 *homologs, *DDX3X *and *DDX3Y*, a single autosomal *PL10 *putative homologous sequence is present in each genome of the studied non-rodent eutheria. These autosomal homologous sequences originated from the retroposition of *DDX3X *but were pseudogenized during the evolution. In rodents, besides *Ddx3x *and *Ddx3y*, we found not only *Pl10 *but another autosomal homologous region, both of which also originated from the *Ddx3x *retroposition. These retropositions occurred after the divergence of eutheria and opossum. In contrast, an additional X putative homologous sequence was detected in primates and originated from the transposition of *DDX3Y*. The evolution of *PL10 *homologs was under positive selection and the elevated Ka/Ks ratios were observed in the eutherian lineages for *DDX3Y *but not *PL10 *and *DDX3X*, suggesting relaxed selective constraints on *DDX3Y*. Contrary to the highly conserved domains, several sites with relaxed selective constraints flanking the domains in the mammalian *PL10 *homologs may play roles in enhancing the gene function in a lineage-specific manner.

**Conclusion:**

The eutherian *DDX3X/DDX3Y *in the X/Y-added region originated from the translocation of the ancient *PL10 *ortholog on the ancestral autosome, whereas the eutherian *PL10 *was retroposed from *DDX3X*. In addition to the functional *PL10*/*DDX3X*/*DDX3Y*, conserved homologous regions on the autosomes and X chromosome are present. The autosomal homologs were also derived from *DDX3X*, whereas the additional X-homologs were derived from *DDX3Y*. These homologs were apparently pseudogenized but may still be active transcriptionally. The evolution of *PL10 *homologs was positively selected.

## Background

*PL10 *was first identified in mouse by using a human Y chromosome (Chr) derived probe [1] and is present in a wide range of eukaryotes from yeast, plants, and animals, including humans [2]. In the mouse, *Pl10 *has been shown to encode a functional protein with an important DEAD motif (Asp-Glu-Ala-Asp), which plays essential roles in spermatogenesis [3]. In eutherian mammals, *PL10 *has two closely-related paralogs, *DDX3X (DEAD box polypeptide 3, X-linked) *and *DDX3Y (DEAD box polypeptide 3, Y-linked*), located on the sex Chrs. *PL10, DDX3X *and *DDX3Y *share the DEAD motif and constitute the *DDX3/DED1 *(*ATP-dependent DEAD-box RNA helicase*) subfamily under the DEAD-box helicase family [4] with a major function related to RNA metabolism [5]. The *DDX3/DED1 *subfamily is involved in diverse cellular process including tissue differentiation at distinct developmental stages, embryogenesis, asexual reproduction, cell regeneration, tumorigenesis and immune response [2,6-8], which have been reviewed comprehensively by Rosner *et al*. [2].

Interestingly, the biological roles of the eutherian members in *DDX3 *subfamily appear to be varied and lineage-dependent although they share domain structures and highly similar sequences. In eutheria, the *DDX3X *has been shown to elicit immunoresponse because the *DDX3X *can interact with *TANK-binding kinase 1 *(*TBK1*) to induce the *type I interferon *(*IFN*) promoter and the downstream immune pathway [6]. In addition, *DDX3X *also plays a role in HIV infection and becomes an important target in antiviral therapy [9]. On the other hand, the human *DDX3Y *lies within the *azoospermia factor *a (*AZFa*) region on the proximal Yq11.21 and the deletion of human *DDX3Y *resulted in the oligozoospermia, azoospermia and the male sertoli-cell only syndrome [10,11]. In spite of their high amino acid (aa) similarity (91%), *DDX3X *cannot rescue the loss-of-function of *DDX3Y *in human [2], signifying the functional diversification between *DDX3X *and *DDX3Y*. The human *DDX3Y *is believed to be one of the essential genes involved in human spermatogenesis and male fertility [12]. In contrast to the human ortholog, the pivotal role of *Ddx3y *in spermatogenesis has been replaced by the autosomal *Pl10 *in mice [3]. The mouse *Pl10 *is believed to evolve from *Ddx3x *through the retroposition mechanism [13]. More interestingly, the bovine *PL10 *has also been proved to be active at the transcription level even though it may lose protein-coding potential [14]. In addition to the lineage-dependent functionality of *PL10*, the tissue specificity of *DDX3X*/*DDX3Y *homologs has also been shown to vary in mouse and human [15,3]. The lineage-specific expression patterns and the diverse genomic structures and locations of *PL10 *homologs suggest that the *PL10 *homologs regulate biological process via divergent mechanisms and evolved differently. However, previous studies focused mainly on elucidating the function rather than the evolution of *PL10*, which elicited our interest to investigate the evolutionary history behind *PL10*, *DDX3X*, and *DDX3Y*. Here, we report the results from a phylogenetic analysis of the *PL10 *homologs in 19 different species.

## Results

### The identification of PL10 homologous sequences

To obtain deep insight into *PL10 *evolution, we collected the *PL10 *related genes deposited in NCBI [16] and detected its potential homologs by comparing the mouse *Pl10 *mRNA sequence against the UCSC genome database [17]. In addition to the 22 annotated sequences for *PL10*, *DDX3X *and *DDX3Y*, we identified 15 *PL10 *putative homologous regions (coverage > 50%) in the genomes of mammals (Table 1). These putative homologs occupied the genomes with two major patterns in terms of their spanning size (2~4 and 10~14 Kb). The large-size homologs are located in the sex Chrs containing intron-exon structures, while the small-size ones are mostly autosomal and intronless (Table 1).

**Table 1:** *PL10 *homologs in 19 species.

Species (build version)	Chr	Gene	Homologous region		Span	Accession Number
Human (37.1)	Y	*DDX3Y*	15016838	15030444	14229	NM_004660.3
	X	*DDX3X*	41193484	41207386	14657	NM_001356.3
	X		73340837	73351755	18744	
	4		104493233	104495627	3122	
Chimp (2.1)	Y	*DDX3Y*	18024925	18030276	5352	NM_001008986.1
	X	*DDX3X*	41567709	41578379	10637	ENSPTRT00000048707
	X		73472677	73479677	7000	
	4		106890486	106891981	1496	
Orangutan (2.0.2)	X	*DDX3X*	41920594	41934087	13494	ENSPPYT00000023631
	X		71584239	71585574	1336	
	4		108027398	108029797	2400	
Mouse (37)	Y	*Ddx3y*	599654	615438	15785	NM_012008.1
	X	*Ddx3x*	12858220	12869030	11577	NM_010028.3
	1	*Pl10*	188791295	188794506	3212	NM_033077.2
	1		28046742	28049045	2304	
Rat (RGSC 3.4)	X	*Ddx3x*	21497214	21508627	11414	XM_228701.4
	13	*Pl10*	103083154	103086327	3174	NM_001108858.1*
	19		5498280	5501578	3299	
Dog (2.0)	X	*DDX3X*	35708607	35722852	14282	XM_856175.1
	22		15373292	15375479	2188	
Horse (EquCab2.0)	X	*DDX3X*	33503944	33514664	10721	XM_001491432.2
	17		31837579	31839780	2202	
Cow (Btau_4.0)	Y	*DDX3Y*	86^Δ^	5279^Δ^	5194	[14]
	X	*DDX3X*	68833	82540	13708	[14]
	15	*PL10*	186070	189757	3688	[14]
Opossum (MonDom5)	4	*DDX3*	22331869	22343917	12049	ENSMODT00000026845
Chicken (2.1)	1	*DDX3*	115610539	115617788	7250	NM_001030800.1
X. tropicalis (4.1)		*DDX3*	940555	947979	7425	BC063374
Zebrafish (Zv7)	6	*PL10*	25945	42249	16304	NM_130941
Clamworm		*PL10a*				AM048813.1
Flatworm		*DjVLGA*				AB017002.1
Hydra		*CnPL10*				AB047381.1
Rice		DEAD-box RNA Helicase				NM_001074753.1
Arabidopsis		DEAD-box RNA Helicase				NM_129813.4
Fission Yeast		*DED1*				AJ237697.1
Yeast		*DBP1*				X55993.1
		*DED1*				X57278.1

* The corresponding protein entry is NP_001102328.1.

^Δ ^The position was annotated based on NW_001496707.1.

We extracted the sequences from these putative homologous regions and conducted a gene prediction using GENSCAN [18] to identify whether these homologous sequences maintain protein-coding potential. Based on gene similarity, structures and chromosomal locations, we obtained the predicted DDX3X in chimp and orangutan, and Pl10 on Chr 13 in rat (RNO13). The predicted chimp and orangutan DDX3X matched the predicted coding proteins in ENSEMBL [19] (Table 1). Compared to the human DDX3X protein of 662 aa, the predicted peptide is much shorter in the chimp with only 438 aa because of the incomplete sequence. The predicted rat Pl10 matched to the entry, NP_001102328.1, in the NCBI database, and we concluded that it is the rat *Pl10 *based on its intronless structure and high sequence similarity (96%) with the mouse *Pl10*. In opossum, the predicted DDX3 peptide matched to ENSMODT00000026845 in ENSEMBL [19] (Table 1). The remaining 11 homologs either do not have an open reading frame (ORF) or have a premature stop codon (Table 2). Thus, they are pseudogenes.

**Table 2:** Pairwise comparison between mouse Pl10 (mPl10) and the non-annotated homologous regions in eutheria.

Genomic Position of Non-annotated *PL10 *Homologs		Identity with m *Pl10 *(%)	Alignment Coverage with m *Pl10 *(%)*	Aligned Segment Number^Δ^	Putative Peptide Length (aa)^#^
Human Chr4	(HSA4)	80.08	100.00	1	84
Human ChrX	(HSAX)	78.52	98.38	2 (Ins: 8958 bp)	241(DDX3Y)
Chimp Chr4	(PTR4)	74.83	81.93	1	84
Chimp ChrX	(PTRX)	78.37	98.34	2 (Ins: 1134 bp; Ns: 3010 bp)	472(DDX3X)
Orangutan Chr4	(PPY4)	79.80	74.18	2 (Ns: 463 bp)	132
Orangutan ChrX	(PPYX)	80.94	67.42	1	124(DDX3Y)
Mouse Chr1	(MMU1)	79.45	95.41	1	349(DDX3X)
Rat Chr19	(RNO19)	80.27	51.08	3 (Ns: 1509 bp; Ins: 316 bp; Gap: 962 bp)	192(DDX3X)
Dog Chr22	(CFA22)	73.00	58.00	1	N/A
Horse Chr17	(ECA17)	75.98	96.82	1	N/A
Cow Chr15	(BTA15)	81.00	72.52	1	N/A

* The alignment coverage was calculated based on the pairwise alignment between the mouse *Pl10 *and identified homologous regions.

^Δ ^Ns: the homologous region contains incomplete sequences. Ins: the homologous region is interrupted by non-homologous sequences. Gap: part of m*Pl10 *was not alignable with the detected homologous sequences.

^# ^The peptides were predicted via GENSCAN [18]. The protein name in parenthesis indicated the matched entries with lowest e-value in blastp analysis. N/A: not applied.

### The analyses of PL10 phylogeny

**Figure 1:**
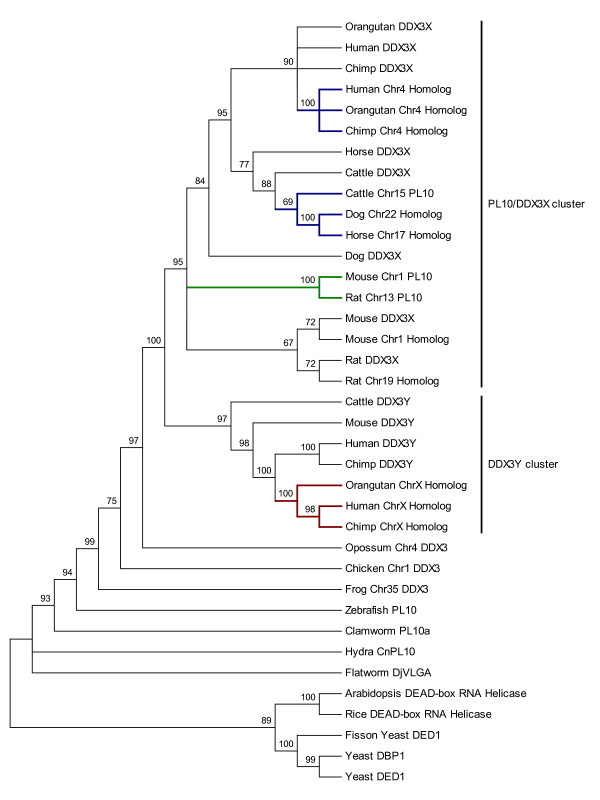
**The bootstrap consensus tree of *PL10 *homologous sequences**. The evolutionary tree was built based on the Neighbor-Joining method implemented in MEGA4 [55,62]. The bootstrap consensus tree is inferred from 1000 replicates and the branches corresponding to partitions reproduced in less than 65% bootstrap replicates are collapsed. The bootstrap values are shown as percentages next to the branches. The evolutionary distances were computed using the Maximum Composite Likelihood method [63] and in the units of the number of base substitutions per site. The rate variation among sites was modeled with a gamma distribution (shape parameter = 0.91). All positions containing alignment gaps and missing data were eliminated by pairwise deletion. A total of 3944 positions were in the final dataset [Additional File 6]. The branches leading to the non-annotated autosomal homologous clusters of *PL10 *are highlighted in blue; the branches leading to the rodent *Pl10 *are highlighted in green; the branches leading to the non-annotated X-homologs are highlighted in red. The *PL10/DDX3X *cluster and the *DDX3Y *cluster are marked by vertical lines on the right.

Using the 22 *PL10 *related entries from NCBI together with 15 previously-described putatively homologous sequences, we constructed a phylogenetic tree to investigate the evolutionary relationship among these homologs. The tree clearly indicated several evolutionary clusters (Fig. 1). The first cluster is the PL10/DDX3X cluster, within which the putative homologous sequences on primate Chr4 were in the same clade and clustered with the primate *DDX3X*. The autosomal homologous regions in ruminants and carnivores, including the bovine *PL10 *pseudogene [14], were also in the same cluster and grouped with the *DDX3X *counterparts as in primates (Fig. 1). No apparent insertions were detected in these homologous regions. The mouse and rat *Pl10 *were in the same branch. However, an additional putative homolog of *DDX3X *was detected in mouse (MMU1) and rat (RNO19), which was grouped with its corresponding *DDX3X *gene, respectively, before clustering them together into a single group. It is noteworthy that all homologs of *DDX3X *identified in mammals are intronless (Table 1). Since the mammalian *DDX3X *contains an intron-exon structure, we reasoned that these intronless homologs are most likely the evolutionary trace after the *DDX3X *retroposition.

In addition to the functional eutherian *DDX3X*, we detected another putative homologous region on primate ChrX which was present on the same branch with the *DDX3Y *instead of the *DDX3X *(Fig. 1). In contrast to the autosomal homologs, these ChrX putative homologs contain one or more insertions that appear to fit with the typical GT/AG splicing rule (Table 2), raising the possibility that these additional X homologs may have derived from a transposition event before the primate divergence (Fig. 1). Furthermore, in opossum and chicken, only a single homologous region with the intron-exon structure was detected on opossum Chr4 (MDO4) and chicken Chr1 (GGA1), respectively (Table 1).

### Positive selection test for the PL10 related genes

**Figure 2:**
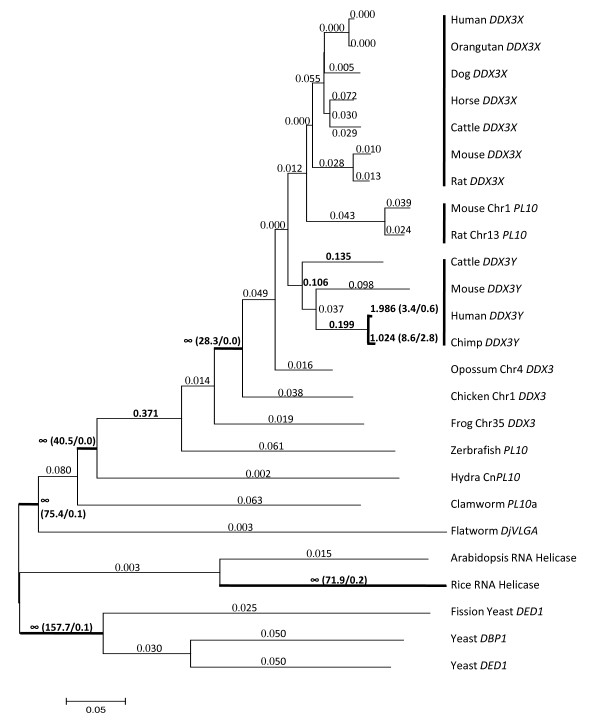
**The tree of the *DDX3 *family established for the positive selection test based on Maximum Likelihood approach**. The branch length was estimated in the unit of the number of nucleotide substitutions per nucleotide. Values larger than 0.1 are denoted in bold. The numbers in the parenthesis represent the estimated numbers of nonsynonymous substitutions against synonymous substitutions of the specific branch. Scale bar = 0.05 unit.

We compared the one-ratio model with the free-ratio model to test the lineage-specific positive selection for the *PL10 *related functional homologs in our dataset using PAML4 package [20]. The one-ratio model assumes the same K_a_/K_S _(*w*) ratio for all the lineages. The log-likelihood value under this model was *l*_0 _= -22217.097 with 58 parameters where the transition/transversion rate ratio was *k *= 1.685 and *w *= 0.041. The *w *was computed as the average of all codon sites and lineages. The free-ratio model assumes an independent *w *ratio for each branch and the number of parameters was increased to 104 for our dataset in this model. The likelihood value under this model was *l*_1 _= -22105.980. The comparison of the likelihood value, 2Δ*l *= 2(*l*_1_-*l*_0_), was 222.234 as determined by the *X*^2 ^distribution with degree of freedom (df) of 46 (p < 0.001), allowing us to reject the one-ratio model and conclude that the *w *ratios are varied among lineages (Fig. 2). In mammals, the estimates of *w *ratios were all lower than 0.1 on the branches leading to the lineages for *PL10 *and *DDX3X*, whereas the *w *ratios were higher on average (0.5) among the lineages for *DDX3Y *(Fig. 2). Furthermore, the primate lineages for *DDX3Y *in human and chimp were detected to be subject to positive selection (Fig. 2). The branches leading to the mammalian *PL10 *homologs clade (Fig. 2) also showed *w *ratios larger than 1, suggesting that the evolution of *PL10*/*DDX3X/DDX3Y *was under positive selection.

**Figure 3:**
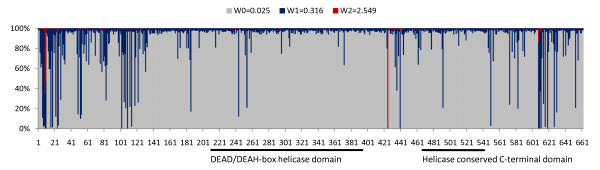
**Posterior probabilities of three site classes with different selective pressures (measured by the *w *ratio) for codon sites along the mammalian PL10 homologs under the site model M3**. The X-axis represents the codon positions which were labeled based on the human DDX3X amino acids. The probabilities of the site classes are indicated in the Y-axis. The DEAD/DEAH-box helicase domain and helicase conserved C-terminal domain are underlined.

Since some lineages were positively selected, especially in the case of *DDX3Y*, we further used a small dataset containing only the mammalian homologous coding sequences to examine the positively selected sites. The test statistic of likelihood ratio test (LRT) between the one-ratio model (M0) and the discrete model (M3) was 128.182 that is greater than the critical value  = 13.28 when df = 4 [Additional File 1 and File 2]. This suggested that the selective pressure is diverse among the codons. Three site classes calculated under model M3 have prior probability of p_0 _= 0.887, p_1 _= 0.108, and p_3 _= 0.005 with the Ka/Ks ratios of *w*_0 _= 0.025, *w*_1 _= 0.316 and *w*_2 _= 2.549 [Additional File 1]. The posterior probabilities of site classes calculated in model M3 are shown in Fig. 3. However, the LRT of the other two pairs of models, M1a (Nearly Neutral)/M2a (Selection) and M7 (beta)/M8 (beta & w), generated an incongruent result. The test statistic of the M1a/M2a is insignificant (p > 1), whereas the M7/M8 generated a significant result with a LRT value of 16.97 greater than the critical value at df = 2,  = 9.1 (p < 0.01) [Additional File 1 and File 2], which together gave rise to the marginal prediction of the codon sites with relaxed selective constraints. Four (9A, 10L, 24S, 425S) and six (9A, 10L, 24S, 425S, 608A, 609S) sites were inferred to contain increased *w *ratios under models M2a and M8, respectively. Four of the six inferred sites in model M8 coincided with the result of model M2a, including Ala9, Leu10, Ser24, Ser425, in which the Ser24 and Ser425 have posterior probability higher than 0.9 under model M8 [Additional File 1]. All of the inferred sites are located in the non-domain regions.

### Conservation of PL10 homologs

**Figure 4:**
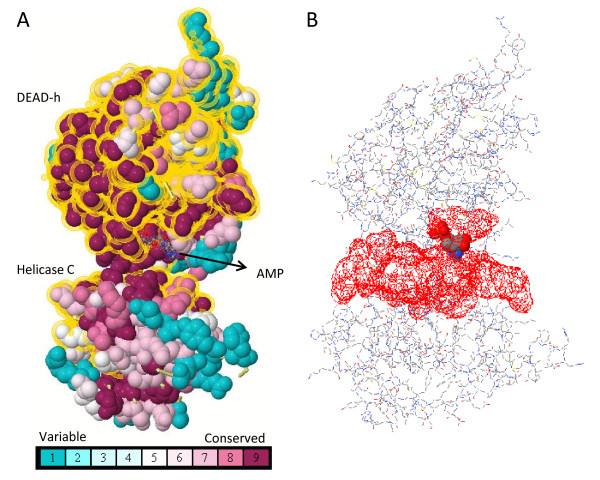
**The conserved domain and ATP binding site of the human DDX3X**. **A**. The conservation score distribution on the human DDX3X (PDB: 2I4I) was assigned based on the empirical Bayesian method by ConSurf [21]. The domain regions are highlighted in dot-yellow halos. **B**. The ATP binding cleft depicted in PDBsum [64] corresponds to the conserved region in A.

We conducted a multiple alignment for all the analyzed sequences to investigate the domain conservation in *PL10*, *DDX3X *and *DDX3Y*, and found that the DEAD/DEAH box helicase domain (Pfam: PF00270) and helicase conserved C-terminal domain (Pfam: PF00271) of the *DDX3 *genes are highly conserved [Additional File 3]. We evaluated the degree of conservation by ConSurf (Fig. 4A) [21], which assigned the conservation score to each site of the provided DDX3X structures (PDB: 2I4I) [22] based on the empirical Bayesian method [21]. After mapping the conservation score to the structure, we found that the highly conserved codons concentrated in the cleft where the adenosine monophosphate (AMP) and RNA substrates interact with the DDX3 proteins (Fig. 4).

## Discussion

In the non-eutherian lineages, *PL10 *is the sole member of the *DDX3 *subfamily, whereas in eutheria, the ancient *PL10 *gene is located on the ancestral sex Chrs, resulting in the sex Chr-linked orthologs, *DDX3X *and *DDX3Y *(Fig. 1). Molecular evolutionary studies in recent years have established that the mammalian sex Chrs originated from a pair of ordinary autosomes, and most ancestral genes on that pair were still maintained on the X Chr but degenerated on the Y Chr due to the lack of recombination [23-25]. However, the Y Chr intends to maintain the functional genes that are beneficial to the male, such as those genes involved in spermatogenesis including *DDX3Y *[26]. Like the non-eutherian *PL10*, *DDX3X *and *DDX3Y *comprise the intron-exon structures, supporting the concept that *DDX3Y *and *DDX3X *are the evolutionary relics of the ancestral autosomal *PL10*. In opossum, the *PL10 *homologous sequence was detected only on MDO4 but not on the sex Chrs. The opossum Chr4 homolog also contains the intron-exon structure with predicted peptide close to *DDX3X*. Similarly, the single homologous sequence detected in chicken was located on the autosomes and it contains introns. A recent study for the gene cluster in the X/Y-added region of mammalian sex Chrs, XAR and YAR, revealed that the gene cluster and the gene order of this region are the same on chicken GGA1 but separated on opossum MDO4 and MDO7 [15], suggesting a single translocation event gave rise to the different chromosomal locations of the gene cluster among chicken and opossum. The *DDX3X/DDX3Y *also reside within the XAR/YAR, which allowed us to reach the parallel conclusion that the translocation generated the *PL10 *homologs on the chicken Chr1 and opossum Chr4, and *DDX3X/DDX3Y *on the eutherian sex Chrs (Table 1, Fig. 1).

Of particular interest is the occurrence of the mouse *Pl10*, an intronless gene and the only demonstrated functional autosomal ortholog in mammals to date. Consistent with a previous deduction [13], our result supported that the rodent *Pl10 *was derived from the retroposition of the *DDX3X *genes [27]. Retroposition is a crucial mechanism of gene duplication [28] and generates many new genes in new genomic positions through the reverse transcription of a parental gene [27,29,30]. The parental gene usually contains introns, whereas the processed retrocopy is intronless [27]. Thus, the other detected putative autosomal *PL10 *homologous sequences without apparent intron-exon structure in cattle, horse, dog and primates may have also evolved through the retroposition mechanism. These intronless homologous sequences on autosomes were consistently detected in eutherians but not in opossum, suggesting that the retroposition occurred after the divergence of eutherian and other mammals around 150 to 170 million years ago [31]. This raised an interesting question, why does the autosomal retroposition of the *PL10 *occur specifically in eutheria? It may be partially explained by the important functional role of mouse *Pl10 *and the recently discovered bovine *PL10*. The mouse *Pl10 *has been evidenced to be a central gene regulating the spermatogenesis and replace the role of *DDX3Y *[3]. The bovine *PL10*, albeit pseudogenized during the evolution, has also been proven to be active transcriptionally and may be involved in the regulatory coordination of bovine spermatogenesis [14]. Although the coding potential of the autosomal *PL10 *homologous sequences in eutheria, except in mouse, is diminished, we cannot exclude the possibility that these homologous sequences may be involved in regulating some biological process at the transcriptional level. Indeed, previous studies suggested that the pseudogenes may regulate the expression of the functional paralogous genes by producing antisense RNA [32,33]. Therefore, it is valuable to investigate whether these homologous sequences are transcriptable and their potential function in the future.

The maximum likelihood ratio test (LRT) for different lineages indicated that the Ka/Ks ratios in the *PL10 *homologs are varied among the evolutionary lineages. The Ka/Ks ratio along the branches among the mammalian lineages showed that the evolution of the mammalian *PL10 *homologs were not subject to positive selection, except for the human and chimp *DDX3Y *that are positively selected (*w *> 1). In addition to the chimp and human *DDX3Y*, we found that the ratios for the other eutherian lineages for *DDX3Y *appear to be higher when compared to those for mammalian *PL10 *and *DDX3X*, which is in line with a finding by Wilson and Makova [15]. These elevated *w *ratios can be explained by either the effect of relaxed selective constraints for the lineages containing *DDX3Y *due to the absent recombination of the ChrY or a weak positive selection operating on the Y-homologs [20]. The latter may still continue to refine the male-specific function for the Y-homologs [34]. In contrast, the mammalian lineages for *DDX3X *and *PL10 *with extremely low w ratios suggested that purifying selection may act strongly on the mammalian *PL10 *and X-homologs. Furthermore, the *w *ratios of the branches leading to the avian and mammalian lineages were larger than 1, indicating that the emergence of eutherian *PL10 *homologs was selected positively to acquire species-specific gene function and purifying selection acted on the *DDX3X *and *PL10 *homologs to preserve their crucial biological function and avoid their divergence.

A *DDX3X*/*DDX3Y*-specific multi-residue insertion (EALRAMKENG) has been observed to form an important positively charged cavity with the neighboring positive residues to increase the RNA binding surface in humans [35]. After incorporating *PL10 *and the homologous regions, we found that the insertion was highly conserved in the *PL10 *homologs of fish, frog, chicken, and other mammals, suggesting that the functional constraints occurred along the cavity region in the related homologs. Conversely, this insertion was not well-conserved in plants and invertebrates. Furthermore, the *DDX3X *displayed no activity with the RNA substrate when different flanking regions surrounding the domains were removed [35]. A similar effect was proven in other DEAD-box helicase related genes, such as the *UAP56 *[36] and *DP103 *[37], where the deletion of either the N-terminal or C-terminal flanking sequences outside the domain core regions impacts their helicase and ATPase activity, signifying the regulatory roles of the flanking regions. As shown from the analysis of positively selected amino acids, all the marginally inferred selected sites were located in the non-domain regions (Fig. 3) and may have served as the targets for improving the gene function during the evolution. One of the inferred sites, Ser425, occurred in the hinge region between two domains, suggesting its potential role in the adaption of the PL10 protein conformations and ligand-binding specificity. The slightly elevated *w *ratios were observed mostly in the non-domain regions as depicted in Fig. 3, indicating the purifying selection may attenuate in these regions to allow the functional accommodations of the PL10 homologs. Moreover, several sites in the flanking regions of the human DDX3X have been shown to undergo epigenetic modifications, including Ser2[38], Tyr69[39,40], Ser74[41], Ser76[41], Ser78[41], Tyr104[39], Ser125[41], Ser590[42], Ser594[43,42] and Ser612[43]. Meanwhile, two sites in the flanking sequences of the human DDX3Y, Tyr69 [39,44] and Ser592 [45], have been shown to be phosphorylated, and they are conserved with the modified sites at Tyr69 and Ser594 in the human DDX3X. The mouse Pl10 also has two phosphorylated sites at Tyr282 and Tyr465, but both of them were located in the domains [46,47]. The sequence comparison showed that the selection force has limited the divergence in the regions flanking the domains of PL10 related genes in fish, frog, bird and mammals, and most of the epigenetically modified sites were highly conserved among these species. Interestingly, despite the high degree of conservation, Ser76 exists specifically in the PL10 and DDX3X homologs but not in the DDX3Y orthologs. This distinction and different epigenetic modification pattern may partly contribute to the functional divergence between the eutherian *DDX3Y*, *DDX3X *and *PL10*. The functional specificity of *PL10 *homologs appear to be determined multifactorially, including the sequence elements located in the non-conserved regions, the factors controlling the diverse temporal and spatial expression patterns [14], and the distinct epigenetic modification patterns [35].

This study was limited to the availability of complete genomes and the accuracy of the genome assembly. Even though the number of finished genome projects in diverse species is growing, incomplete sequences of *PL10 *homologs still exist in the published genomes, especially for the challenges in sequencing and assembly of the Y Chr due to its highly repetitive nature. Further understanding of the evolution of sex Chr linked genes largely relies on the clarification of the diverse genomes in species other than primates.

## Conclusion

Our analyses revealed that several conserved putative *PL10 *homologous regions, in addition to the functional *PL10*/*DDX3X*/*DDX3Y*, are present on the autosome and mammalian X Chr. These homologs share high similarity (> 70%) and coverage (> 50%) with mouse *Pl10 *but contain premature stop codons or indels, resulting in shorter putative peptides and/or frameshifts, suggesting their pseudogenization during the course of evolution [27,48]. The eutherian *DDX3X/DDX3Y *located in XAR/YAR were derived from the translocation of the orthologs on the ancestral autosome [15]. The identified putative autosomal homologs in mammals in the present study were retroposed from the *DDX3X *while the additional X-homologs in primates were transposed from the *DDX3Y*. These translocation events are lineage-specific. Like the bovine *PL10*, these homologs may still be active transcriptionally. Positive selection appears to operate on the *PL10 *homologs during the evolution. In addition to the highly conserved domain regions, several sites in the non-domain regions of functional *PL10 *homologs may play roles in enhancing the gene function in a lineage-specific manner.

The results reported in this study not only increase our knowledge regarding the molecular evolution of *PL10 *homologs, which will facilitate the future functional characterization of *PL10 *homologs, but also provide a valuable model to investigate the origin and evolution of the mammalian sex Chrs and the mechanisms behind lineage-specific gene duplication and functionality.

## Methods

### Sequence retrieval

By blating [49] the mouse *Pl10 *mRNA sequence against the genomes in UCSC genome database [17], we detected several putative homologous regions in mammals, birds and amphibians. We retrieved the homologous sequences from UCSC and conducted the pairwise alignment by the Bl2seq (NCBI Blast package) program using the mouse *Pl10 *mRNA sequence as the subject to filter out the sequences which cover < 50% of the mouse *Pl10*. We excluded the homologous sequences for the species without clear chromosomal annotations from our study. In addition, we downloaded the *PL10 *homologous sequences for other species from the NCBI nucleotide database based on the literature and database mining [2]. Afterwards, we used blastx [50] to confirm the identity of each sequence and retrieved the corresponding entries from the NCBI when the query sequences matched these entries perfectly. Alternatively, for sequences without a perfect hit in the database, we used the collected genomic sequences for gene prediction using the GENSCAN [18]. The predicted peptides were used for blastp [50] analysis to clarify their identities. Whenever the predicted proteins can match to the entries in the database, we used the deposited sequences in our analyses. The results were summarized in Tables 1 and 2. Several purpose-designed scripts were coded in C++ to facilitate the analysis.

### Phylogenetic analysis

We performed multiple sequence alignments to investigate the conservation of the domain regions by ClustalW [51]. In order to ensure alignment quality, we first pre-aligned the annotated homologous sequences using their translated amino acid sequences in the coding regions. The parameters of the ClustalW for multiple alignment stage were modified to 3.0 for the gap opening penalty and 1.8 for the gap extension penalty to improve the alignment. Following that, we aligned the non-annotated homologous sequences based on the nucleotide sequences, which was further refined by manual adjustment. The positions of DEAD and the helicase domain were defined based on the annotation of Pfam [52] for the human *DDX3X*. The alignment was visualized through Jalview [53]. The degree of conservation was calculated by the empirical Bayesian method implemented in ConSurf [21] to investigate the highly conserved sites in the published human *DDX3X *structures (PDB: 2I4I) covering the important interacting domains involved in the RNA metabolism of the *PL10 *related homologs [35]. The conserved degree was represented through different colors using Jmol [54] as shown in Fig. 4A. In addition, we used the alignment to establish the Neighbor-Joining (NJ) phylogenic tree to study the relationship between *PL10*, *DDX3X*, and *DDX3Y *by MEGA4 [55] with the Maximum Composition Likelihood approach and 1000 bootstrap replicates (Fig. 1). The rate variation among sites was modeled with a gamma distribution (shape parameter = 0.91, estimated by the Model Selection of TOPALi (version 2.5 [56]). In Fig. 1, the branches corresponding to partitions reproduced in less than 65% bootstrap replicates are collapsed. The reason for using the NJ method is that the average pairwise Jukes-Cantor (JC) distance of the dataset is 0.362 smaller than 1.0, which is suitable for making the NJ trees [57]. We applied the pairwise deletion to remove gaps as our sequence lengths are varied, and a complete removal of the gaps is not a good choice as it eliminates a large portion of phylogenetically meaningful sites from consideration. Further, we used the Maximum Composite Likelihood model, recommended by the author of MEGA4 as a better evolutionary model. We also used the PHYML [58] and MrBayes [59] implemented in TOPALi to generate the phylogenetic trees [Additional File 4 and File 5]. The models used in PHYML and MrBayes are general-time-reversible (GTR+Gamma) selected via the TOPALi.

Potential pseudogenes were excluded from the positive selection test since the selective constraint may not act on them anymore. We prepared an amino acid alignment and the corresponding cDNA alignment for the complete set of 25 sequences for the lineage test and a small dataset (14) containing only the functional mammalian *PL10 *homologs for the site-specific test. The Codeml [20] in PAML4 was applied for the following analyses. The sites and lineages subject to positive selection were detected based on the maximum likelihood approach [60]. We compared the log likelihood values (*l*) derived from pairs of models to testify if there was a significant difference between model pairs by LRT. Each pair of the models contains a simple model, where the Ka/Ks ratios of the sites are limited, and a complex model, where the Ka/Ks ratios can be varied. We can infer the occurrence of lineage-specific and site-specific positive selection when the estimated Ka/Ks ratio in the complex model is larger than 1.0 and the calculated test statistic (2Δ*l*) is significantly larger than the critical values of the *X*^2 ^distribution at the corresponding degrees of freedom. We used M0 (one-ratio) and M1 (free-ratio) to test whether the lineage-specific positive selection occurred. The pairs of models used for the site-specific test were M0 (One Ratio)/M3 (Discrete), M1a (Nearly Neutral)/M2a (Positive Selection), M7 (beta)/M8 (beta & w) [20]. Each test was repeated to ensure the reproducible statistical results. The detailed assumptions and descriptions of each model were illustrated by Yang *et al*. [61,20].

## Authors' contributions

TCC assisted in designing the study, collected the data, carried out the analyses and drafted the manuscript. WSL conceived of the study, participated in the experimental design and edited the manuscript. All authors read and approved the final manuscript.

## Supplementary Material

Additional file 1 **Selection test result for functional mammalian *PL10 *homologs**. Log-likelihood and parametric estimates of the site-specific positive selection for functional mammalian *PL10 *homologs. Click here for file

Additional file 2 **Test statistics of site-specific positive selection test**. Likelihood ratio statistics (2Δ*l*) of the site-specific positive selection test. Click here for file

Additional file 3 **The multiple alignment and conserved regions of the PL10 related sequences**. Only the domain regions are shown in this figure. The newly identified homologs are shaded in grey while the DEADc and Helicase C-terminal conserved domain are boxed in red. The blue color residues indicate the sites with conserved identity over 85%. The DDX3X/Y and PL10 specific insertion and the extended DDX3 unique positive residues are highlighted in green. Click here for file

Additional file 4 **The Maximum-likelihood tree built for the *PL10 *related homologous sequences**. The evolutionary tree was built based on the Maximum-likelihood method implemented in TOPALi [56]. The bootstrap values (1000 replicates) are shown next to the branches. The evolutionary model used was GTR+G. The tree has a similar topology to Fig. 1. Compared to Fig. 1, a swap occurred between the branches leading to the hydra, clamworm and flatworm homologs, and another swap observed between the branches leading to the putative rat Chr19 homologous region and other rodent homologous regions. The branches leading to the non-annotated autosomal homologous clusters of *PL10 *in primate are highlighted in blue; the branches leading to the rodent *Pl10 *are highlighted in green; the branches leading to the non-annotated X-homologs are highlighted in red. The *PL10/DDX3X *cluster and the *DDX3Y *cluster are marked by vertical lines on the right. Click here for file

Additional file 5 **The Bayesian inference tree built for the *PL10 *related homologous sequences**. The evolutionary tree was built based on the Bayesian inference method implemented in TOPALi [56]. The bootstrap values (1000 replicates) are shown next to the branches. The evolutionary model used was GTR+G. This tree is very much similar to the Supplementary Fig. 2. Click here for file

Additional file 6 **The multiple sequence alignment of the putative *PL10 *homologs**. A total of 37 sequences from 19 species were included in the alignment. The alignment length is 3944 bp. The number of phylogenetic informative sites is 2954 (79.4%). Click here for file
