# Supplementary material for: The molecular evolution of PL10 homologs
Source: BMC Evol Biol. 2010 May 3;10:127. doi: 10.1186/1471-2148-10-127 (PMC2874800; doi:10.1186/1471-2148-10-127)
Supplement: Additional file 1 — Selection test result for functional mammalian PL10 homologs. Log-likelihood and parametric estimates of the site-specific positive selection for functional mammalian PL10 homologs. [file 1471-2148-10-127-S1.DOC]

**Additional File 1.** Selection test result for functional mammalian *PL10* homologs.

| Model | *p* | ln*L* | *K_a_/K_S_* | Parameters | Positively Selected Sites* |
| --- | --- | --- | --- | --- | --- |
| One Ratio(M0) | 1 | -7773.043 | 0.058 | w=0.058 | None |
| Nearly Neutral(M1a) | 2 | -7720.758 | 0.073 | P_0_=0.965, w_0_=0.040  P_1_=0.035, w_1_=1.000 | Not Allowed |
| Positive Selection(M2a) | 4 | -7720.758 | 0.073 | P_0_=0.965, w_0_=0.040  P_1_=0.035, w_1_=1.000  P_2_=0.000, w_2_=74.326^£^ | 9A, 10L, 24S, 425S |
| Discrete(M3) | 5 | -7708.952 | 0.069 | P_0_=0.887, w_0_=0.025  P_1_=0.108, w_1_=0.316  P_2_=0.005, w_2_=2.549 | 9A, 10L, **24S, 425S** |
| Beta(M7) | 2 | -7718.937 | 0.063 | P= 0.227, q= 3.130 | Not Allowed |
| Beta&w(M8) | 4 | -7710.451 | 0.069 | p_0_=0.994, p=0.292, q= 4.654  p_1_= 0.006, w= 2.344 | 9A, 10L, **24S**, **425S**, 608A, 609S |

The chimp *DDX3X* was removed due to the incomplete sequence. Sites inferred under positive selection at the 90% level are listed in bold. p is the number of parameters in the *w* distribution. The number in column Ka/Ks is the average value over codons.

* Sites were numbered based on the human DDX3X.

£ No sites have been assigned into this class.
