# Supplementary material for: The molecular evolution of PL10 homologs
Source: BMC Evol Biol. 2010 May 3;10:127. doi: 10.1186/1471-2148-10-127 (PMC2874800; doi:10.1186/1471-2148-10-127)
Supplement: Additional file 2 — Test statistics of site-specific positive selection test. Likelihood ratio statistics (2Δl) of the site-specific positive selection test. [file 1471-2148-10-127-S2.DOC]

**Additional File 2.** Test statistics of site-specific positive selection test

| Model Comparison | 2Δ*l* | df | 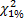 |
| --- | --- | --- | --- |
| M0/M3 | 128.18 | 4 | 13.28 |
| M1a/M2a | 0.00 | 2 | 9.1 |
| M7/M8 | 16.97 | 2 | 9.1 |
