# Supplementary figures and images for: The molecular evolution of PL10 homologs
Source: BMC Evol Biol. 2010 May 3;10:127. doi: 10.1186/1471-2148-10-127 (PMC2874800; doi:10.1186/1471-2148-10-127)

## Slide 1
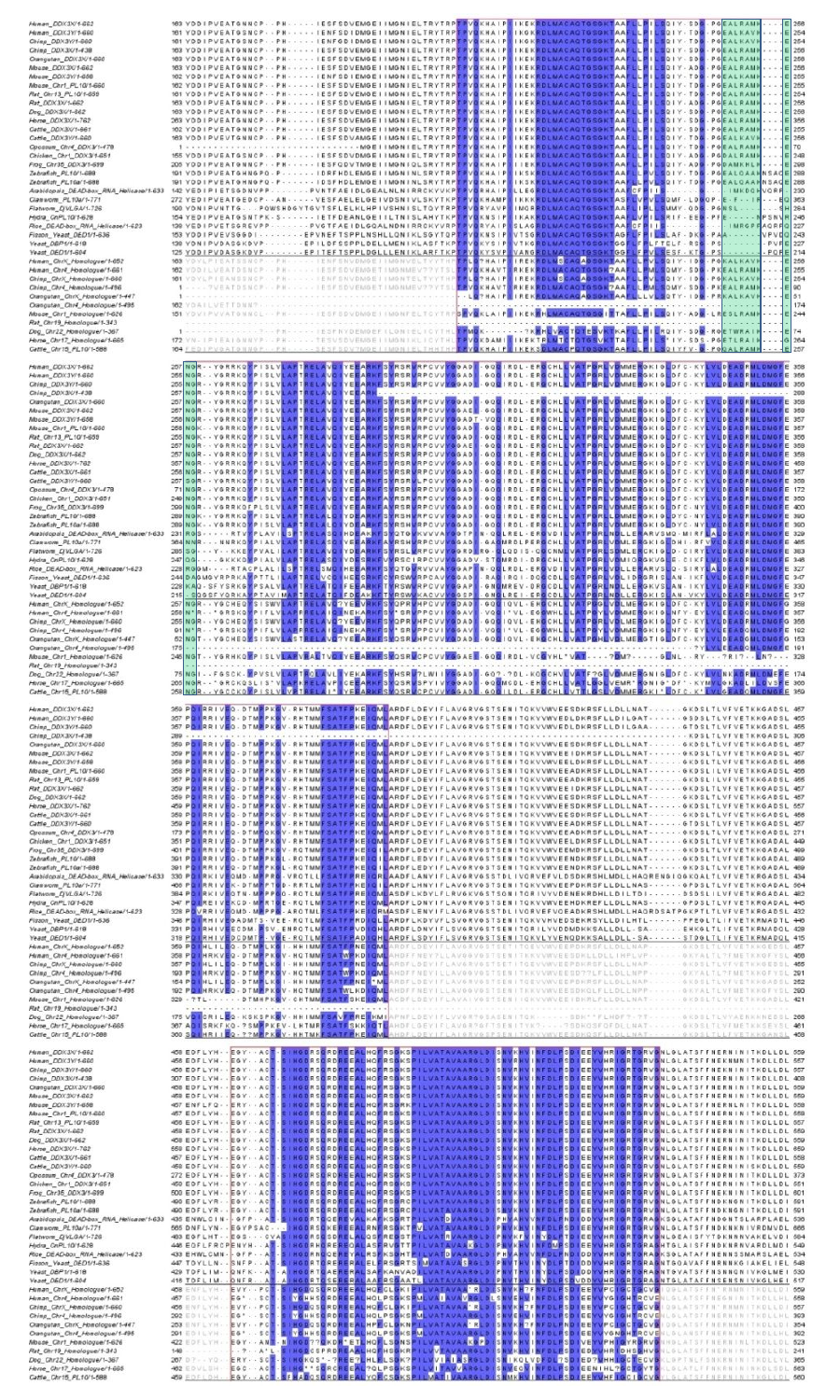

Supplement: Additional file 3 — The multiple alignment and conserved regions of the PL10 related sequences. Only the domain regions are shown in this figure. The newly identified homologs are shaded in grey while the DEADc and Helicase C-terminal conserved domain are boxed in red. The blue color residues indicate the sites with conserved identity over 85%. The DDX3X/Y and PL10 specific insertion and the extended DDX3 unique positive residues are highlighted in green. [file 1471-2148-10-127-S3.PPT]

## Slide 1
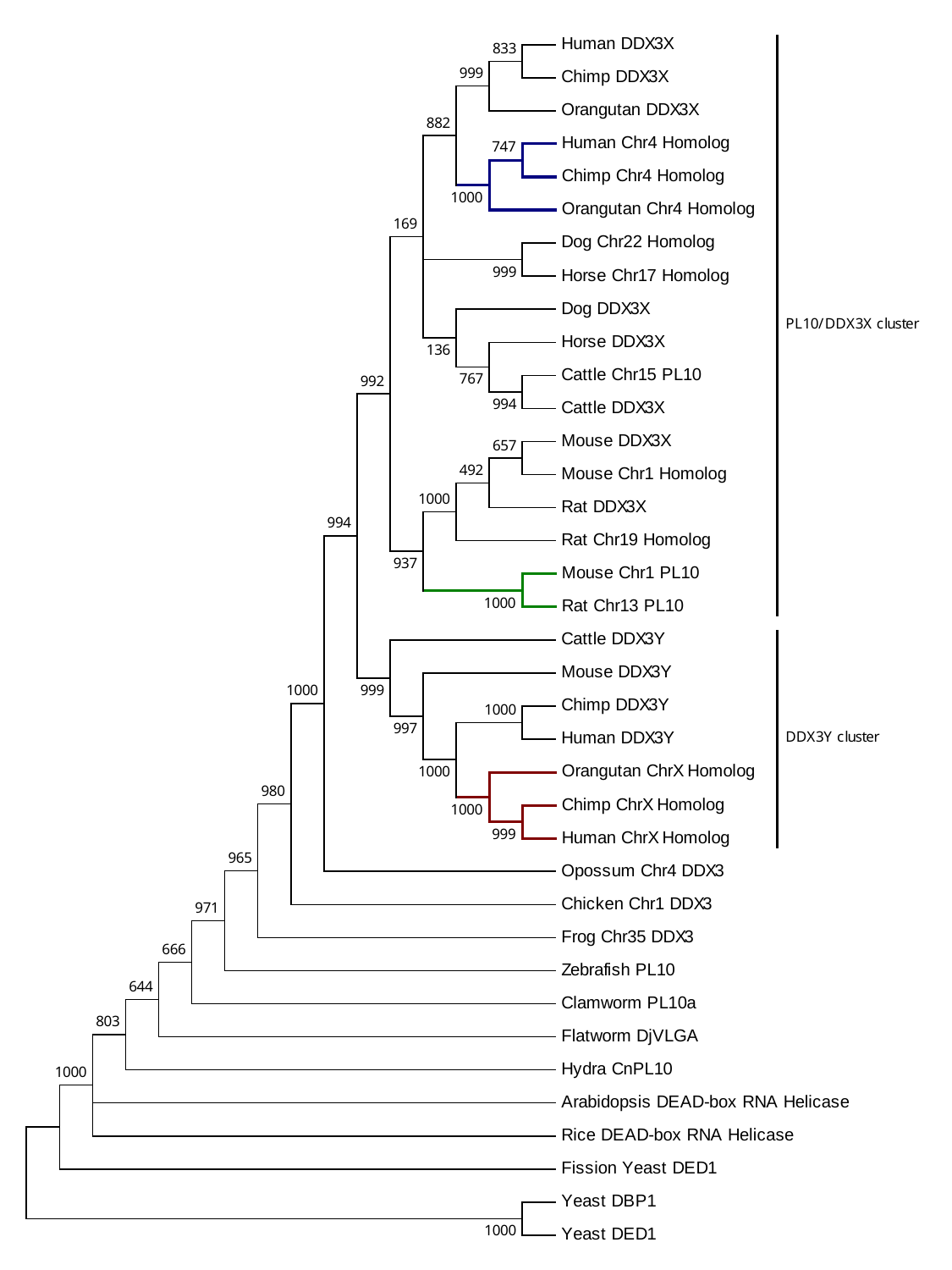

Supplement: Additional file 4 — The Maximum-likelihood tree built for the PL10 related homologous sequences. The evolutionary tree was built based on the Maximum-likelihood method implemented in TOPALi [56]. The bootstrap values (1000 replicates) are shown next to the branches. The evolutionary model used was GTR+G. The tree has a similar topology to Fig. 1. Compared to Fig. 1, a swap occurred between the branches leading to the hydra, clamworm and flatworm homologs, and another swap observed between the branches leading to the putative rat Chr19 homologous region and other rodent homologous regions. The branches leading to the non-annotated autosomal homologous clusters of PL10 in primate are highlighted in blue; the branches leading to the rodent Pl10 are highlighted in green; the branches leading to the non-annotated X-homologs are highlighted in red. The PL10/DDX3X cluster and the DDX3Y cluster are marked by vertical lines on the right. [file 1471-2148-10-127-S4.PPT]

## Slide 1
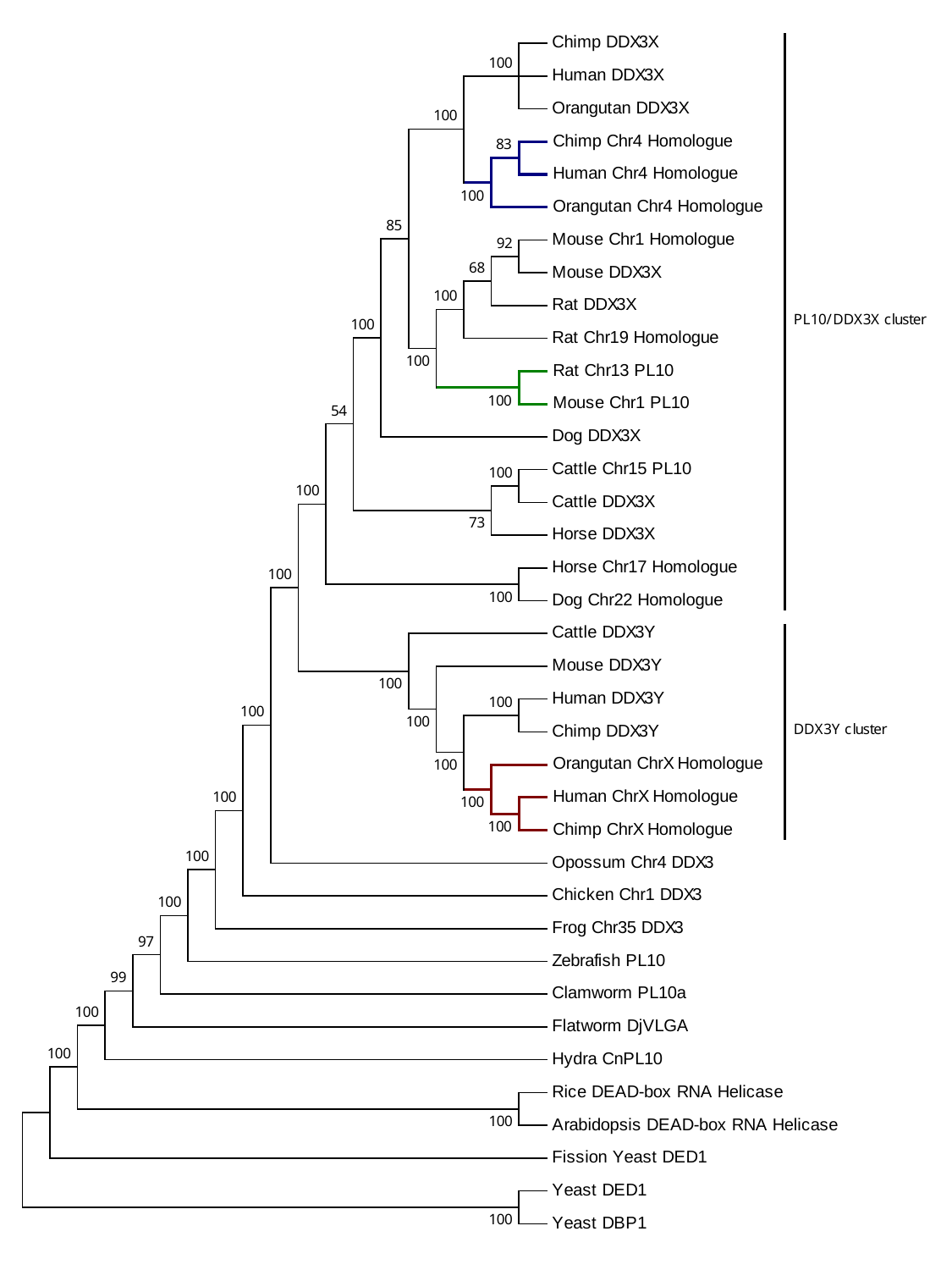

Supplement: Additional file 5 — The Bayesian inference tree built for the PL10 related homologous sequences. The evolutionary tree was built based on the Bayesian inference method implemented in TOPALi [56]. The bootstrap values (1000 replicates) are shown next to the branches. The evolutionary model used was GTR+G. This tree is very much similar to the Supplementary Fig. 2. [file 1471-2148-10-127-S5.PPT]
